# *Parkin* is the most common causative gene in a cohort of mainland Chinese patients with sporadic early‐onset Parkinson's disease

**DOI:** 10.1002/brb3.1765

**Published:** 2020-07-16

**Authors:** Yanyan Jiang, Meng Yu, Jing Chen, Hong Zhou, Wei Sun, Yunchuang Sun, Fan Li, Luhua Wei, Elmar H. Pinkhardt, Lin Zhang, Yun Yuan, Zhaoxia Wang

**Affiliations:** ^1^ Department of Neurology Peking University First Hospital Beijing China; ^2^ Department of Neurology Ulm University Ulm Germany; ^3^ Department of Neurology UC Davis Medical Center Sacramento CA USA

**Keywords:** genetic, next‐generation sequencing, *Parkin*, Parkinson's disease

## Abstract

**Introduction:**

Genetic mutations associated with early‐onset Parkinson's disease (EOPD) vary widely among different ethnicities. We detected the genes associated with EOPD in a Chinese cohort using next‐generation sequencing (NGS) combined with multiplex ligation‐dependent probe amplification (MLPA) and analyzed the phenotypic characteristics of the mutation carriers.

**Methods:**

Cohort of 23 sporadic EOPD patients (onset age ≤ 45 years) were recruited. Genetic causes were identified by a targeted NGS panel containing 136 known extrapyramidal disease‐causative genes. Multiplications or deletions of PD‐causing genes were detected using the MLPA method. Demographic and clinical data were obtained, analyzed, and compared between patients with and those without *Parkin* gene variants.

**Results:**

We identified 14 pathogenic or likely pathogenic variants (12 in *Parkin*, 1 in *LRRK2*, and 1 in *VPS13C*) in 10 patients (43.5%) and 8 rare variants of uncertain significance in 9 patients (39.1%). *Parkin* (34.8%) was the most common causative gene among our patients cohort, and exon deletion (62.5%) was the main type of variant. Patients with *Parkin* mutations had a younger age of onset, longer delay in diagnosis, slower disease progression, higher frequency of hyperreflexia, fatigue, and less hyposmia compared to patients without *Parkin* mutations.

**Conclusion:**

Our results revealed a higher prevalence of *Parkin* mutations in Chinese sporadic EOPD patients, and notably, exon deletion was the most common type of mutation. EOPD patients with *Parkin* mutations showed unique clinical characteristics.

## Graphical abstract

We detected the genes associated with early‐onset Parkinson's disease (EOPD) in a Chinese cohort using next‐generation sequencing combined with multiplex ligation‐dependent probe amplification and analyzed the phenotypic characteristics of the mutation carriers. Our results revealed a higher prevalence of Parkin mutations in Chinese sporadic EOPD patients and notably, exon deletion was the most common type of mutation. EOPD patients with Parkin mutations showed unique clinical characteristics.

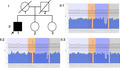

## 1 INTRODUCTION

Parkinson's disease (PD) is the second most common neurodegenerative disease. It is characterized by bradykinesia, rest tremor, rigidity, postural instability, and a progressive loss of dopaminergic neurons. Even though approximately 5%–10% of PD cases have genetic association (Arkinson & Walden, 2018; Lunati, Lesage, & Brice, 2018), the early‐onset PD (EOPD) has a significantly higher probability of finding a genetic cause (Lunati et al., 2018). Previous studies have reported various causative genes in patients with EOPD with diverse detection rates ranging from 3.7% to 16.6% (Youn et al., 2019). Multigene panel tests and whole‐exome sequencing might be effective in identifying genetic variants in Chinese patients with EOPD as shown previously among patients from Korea (Youn et al., 2019), Germany (Trinh et al., 2019), India (Pandey, Tomar, Kumar, Dinesh, & Thelma, 2019), Finland (Siitonen et al., 2017), and United Kingdom (Sandor et al., 2017) and so on. Several studies have reported novel heterozygous mutation in *Parkin* gene using target region capture and high‐throughput sequencing in a single Chinese family with EOPD (Fang, Mao, Zhu, & Li, 2019; Huang et al., 2019; Shi et al., 2018). In recent findings, a clinical and genetic study of early‐onset and familial Parkinsonism in Taiwan including on 324 patients with EOPD and 247 Parkinsonism pedigree provides a better understanding of the genetic architecture of PD in eastern Asia and broadens the clinical spectrum of PD‐causing mutations (Lin, Chen, et al., 2019). Recently, domestic scholars provide a better understanding of the genetic architecture of PD among ethnic Chinese using whole‐exome sequencing(Li et al., 2020). Nevertheless, the research subjects and detection methods of these studies were heterogeneous. In order to provide more data characterizing the genetic basis of mainland Chinese EOPD, we performed next‐generation sequencing (NGS) and multiplex ligation‐dependent probe amplification (MLPA) in 23 Chinese patients with sporadic EOPD.

*Parkin* (PARK2; OMIM#600116) encodes the E3 ubiquitin ligase, which plays an important role in mitochondrial quality control and turnover. Numerous variants throughout *Parkin* are linked to autosomal recessive PD (Arkinson & Walden, 2018). *Parkin* mutations are the most common cause of recessively inherited monogenic PD, accounting for about 8.6% of PD cases with disease onset below the age of 45 years (Kilarski et al., 2012). Younger age, lack of tremor, prominent gait impairment, and the involvement of lower extremities at the time of disease onset were the main characteristics. In addition, a delayed diagnosis was more frequently reported in patients with *Parkin*‐related EOPD (Borsche et al., 2019; Ruiz‐Lopez et al., 2019). Indeed, in the absence of genetic testing results, it is difficult to distinguish *Parkin*‐PD patients from EOPD patients without monogenic cause solely on clinical grounds (Borsche et al., 2019). To better depict the profile of *Parkin*‐PD patients, in the present study, we performed a comprehensive clinical evaluation of EOPD patients and compared patients with and without *Parkin* mutations.

## 2 MATERIALS AND METHODS

### 2.1 Subjects and clinical assessments

Unrelated patients, from outpatient and ward, with EOPD from September 2018 to October 2019 were recruited. The diagnosis of PD was based on the UK Brain Bank diagnostic criteria, and the patients with age of onset less than 45 years were categorized under EOPD. The study was approved by the Ethical Committees of Peking University First Hospital, and an informed written consent was taken from all the participants.

We obtained basic demographic and clinical data, including sex, age, age of onset, disease duration, family history, and current medication. Unified Parkinson disease rating scale (UPDRS) and Hoehn and Yahr (H‐Y) stage were used for the clinical evaluation of both motor and nonmotor features during “off” state. Mini‐mental Status Examination (MMSE) was used for memory and cognitive assessment. We evaluated rapid eye movement (REM) sleep behavior disorder (RBD) using the RBD single‐question screen (RBDSQ). Patients with scores greater than 5, based on RBDSQ, were categorized as having probable RBD (Stiasny‐Kolster et al., 2015). Olfaction testing was performed using the China–Germany version Sniffin’ Sticks’ test (Pinkhardt et al., 2019). All the patients completed clinical evaluation, except one. Levodopa equivalent daily dose (LEDD) was calculated by an established dose equivalence method (Tomlinson et al., 2010).

### 2.2 Genetic tests

Genomic DNA was extracted from patients’ peripheral blood samples using standard procedures, and NGS was performed. Genetic analysis, using a designed gene panel covering 136 most prevalent extrapyramidal disease‐associated genes, was performed (Additional file 1: Table S1). Sanger sequencing with specific primers was performed to confirm the variants detected by NGS. We further performed the MLPA assay (MRC‐Holland, Amsterdam, The Netherlands) to detect exon rearrangements in causative genes, including *DJ‐1*, *ATP13A2, PINK1, UCHL1, SNCA, Parkin*, *LRRK2,* and *GCH1*. Variants were described according to the Human Genome Variation Society (HGVS) nomenclature using nucleotide and amino acid numbering based on published coding DNA reference sequences and protein reference sequences. Then, variants with population frequency over 1% in the dbSNP v137, Exome Variant Server, 1000 Genome, and in‐house Chinese database were filtered out, and only variants predicted to affect the coding regions (including nonsynonymous coding regions, splice acceptor and donor sites, and insertions or deletions [NS/SS/InDel]) were selected for further analysis. Additionally, variants were correlated with patients' phenotypes. Each novel sequence variant was classified as pathogenic, likely pathogenic, variant of uncertain significance, likely benign, or benign according to the 2015 American College of Medical Genetics and Genomics and Association for Molecular Pathology (ACMG‐AMP) guidelines (Richards et al., 2015). Pedigree analysis of the variants was performed among the available family members.

### 2.3 Statistical analysis

Descriptive and analytical statistics methods were applied using software SPSS 25.0. Data were presented as mean ± standard deviation (*SD*). Demographic data and clinical characteristics were compared between EOPD patients with or without *Parkin* gene variants. Quantitative data such as age at onset and disease duration was analyzed by Student's *t* test. Frequency data was compared between groups by chi‐square (*χ* ^2^) test or Fisher exact probability test. *p* < .05 was considered statistically significant.

## 3 RESULTS

### 3.1 Patient characteristics

Unrelated sporadic EOPD patients (23), with 35.48 ± 8.41 years being the mean age at onset and 7.48 ± 6.10 years as mean disease duration, were recruited into this study. Demographic and clinical variables of the subjects are shown in (Additional file 2: Table S2). On evaluating the motor symptoms, 10 patients (43.5%) showed tremor and dyskinesia, while 9 patients (39.1%) showed hyperreflexia. In terms of nonmotor symptoms, 34.8% of enrolled patients had fatigue, 47.8% had constipation, and 8.7% demonstrated RBD. None of the EOPD patients suffered from cognitive impairment. All the patients responded well to levodopa but 43.5% were complicated by peak‐dose dyskinesia. Six patients underwent bilateral subthalamic nucleus deep brain stimulation (DBS), all with good surgical outcomes.

### 3.2 Genetic results

NGS was performed in all of 23 unrelated EOPD patients (Table 1). After identification, 22 variants (12 in *Parkin*, 3 in *LRRK2*, 2 in *VPS13C*, 1 in *DNAJC13*, 1 in *CHCHD2*, 1 in *FBXO7*, and 2 in *GBA*) were detected in 17 participants (17/23, 73.9%). It is noteworthy that exon dosage variation in *Parkin* gene initially suspected in 6 patients by NGS was later confirmed by MLPA method. According to the ACMG guidelines, 14 pathogenic or likely pathogenic variants (12 in *Parkin*, 1 in *LRRK2*, and 1 in *VPS13C*) were detected in 10 patients (10/23, 43.5%), and 8 rare variants of uncertain significance (2 in *LRRK2*, 1 in *VPS13C*, 1 in *DNAJC13*, 1 in *CHCHD2*, 1 in *FBXO7*, and 2 in *GBA*) were detected in 9 patients (9/23,39.1%). Additionally, 2 patients (8.70%) had susceptibility genes *GBA* variants (p. F252I and p. L422R). Furthermore, and remarkably, several novel heterozygous variants were identified, including 1 with p.I1255V in *DNAJC13*, 1 with p.Q33H in *CHCHD2*, 1 with p.T3088P and 1 with p.K926R in the *VPS13C* gene.

**TABLE 1:** Genetic information of patients with EOPD genes carrying variants of uncertain significance and known mutations

ID	Sex	AOO(year)	Gene	cDNA position	AF in gnomAD	Effect on protein	Type of variants	Variants Pathogenicity	Cis/trans test
1	F	19	*Parkin*	Ex6del*, Ho	–	–	Deletion	Pathogenic	Yes
2	M	30	*Parkin*	c.920delT	0	p.L307Rfs*128	Frameshift	Likely Pathogenic	Yes
				c.634T > C	0	p.C212R	Missense	Likely Pathogenic	
3	M	34	*Parkin*	Ex2del*, Ht	–	–	Deletion	Pathogenic	Yes
				Ex3−4del*, Ht	–	–	Deletion	Pathogenic	
4	F	37	*LRRK2*	c.7153G > A*	0.001711	p.G2385R	Missense	VUS	NA
			*DNAJC13*	c.3763A > G	0	p.I1255V	Missense	VUS	
5	M	33	*Parkin*	Ex5−6del*, Ht	–	–	Deletion	Pathogenic	Yes
				Ex6del*, Ht	–	–	Deletion	Pathogenic	
			*CHCHD2*	c.99G > C	0	p.Q33H	Missense	VUS	NA
7	F	35	*LRRK2*	c.6055G > A*	0.000526	p.G2019S	Missense	Pathogenic	NA
8	M	39	*LRRK2*	c.1256C > T*	0.000486	p.A419V	Missense	VUS	NA
9	M	13	*Parkin*	Ex3del*, Ht	–	–	Deletion	Pathogenic	Yes
				c.850G > C*	0.000012	p.G284R	Missense	Pathogenic	
10	F	45	*LRRK2*	c.7153G > A*	0.001711	p.G2385R	Missense	VUS	NA
			*VPS13C*	c.9262delA	0	p.T3088Pfs*25	Frameshift	Likely Pathogenic	
11	M	43	*GBA*	c.754T > A*	0.000044	p.F252I	Missense	VUS	NA
12	F	31	*LRRK2*	c.2264C > T*	0.000741	p.P755L	Missense	VUS	NA
			*FBXO7*	c.155A > G*	0.00041	p.Y52C	Missense	VUS	
15	F	42	*GBA*	c.1265T > G*	0	p.L422R	Missense	VUS	NA
16	F	36	*LRRK2*	c.7153G > A*	0.001711	p.G2385R	Missense	VUS	NA
19	M	31	*Parkin*	Ex5−9del*, Ht	–	–	Deletion	Pathogenic	Yes
				c.634T > C	0	p.C212R	Missense	Likely Pathogenic	
20	F	22	*Parkin*	Ex3del*, Ht	–	–	Deletion	Pathogenic	Yes
				Ex6del*, Ht	–	–	Deletion	Pathogenic	
22	M	35	*Parkin*	c.97C > T*	0.000032	p.R33	Nonsense	Pathogenic	Yes
				c.850G > C*	0.000012	p.G284R	Missense	Pathogenic	
23	M	42	*VPS13C*	c.2777A > G	0	p.K926R	Missense	VUS	NA

Abbreviations: AF, allele frequency; AOO, age of onset; del, deletion; dup, duplication; EOPD, early‐onset Parkinson's disease; Ex, exon; F, female; gnomAD, Genome Aggregation Database; Ho, homozygous; Ht, heterozygous; M, male; NA, not available; VUS, variants of uncertain significance. *Reported variants.

Notably, *Parkin* is the most common causative gene among our patients cohort. Since all eight patients with *Parkin* variants were sporadic, we analyzed whether the two variants were biallelic. Blood samples of the family members of five sporadic patients with *Parkin* variants (patients 2, 3, 9, 20, and 22) were taken for genetic testing to do cis/trans test. In patients 9 and 20, their fathers and mothers carried one of the variants, and in patients 2, 3, and 22, her sister and her brother carried one of the variants separately, all proving the in trans locations of the variants. The other three patients were considered as carrying biallelic mutation, since patient 1 had homozygous exon 6 deletion of *Parkin*, patient 5 had two heterozygous exon deletions (exon 5–6 and exon 6) and patient 19 had a heterozygous exon 5–9 deletion and a heterozygous point mutation in exon 6. So totally, 8 patients with *Parkin* biallelic variants were identified in this cohort, including 7 with compound heterozygous variants and 1 with homozygous variants. The frequency of types of *Parkin* variants is as follows: exon deletion (62.50%), missense (25.00%), nonsense (6.25%), and frameshift (6.25%) variants. Exon deletions can be found in homozygosis, in compound heterozygosis with another exon deletions, or point mutation.

### 3.3 Comparison of clinical data between patients with EOPD with and without Parkin gene variants

Upon comparing the clinical and demographic data in EOPD patients, with or without *Parkin* gene variants, those with *Parkin* gene variants had a younger age at onset (27.13 ± 8.10 vs. 39.93 ± 4.15), longer diagnostic delay (10.63 ± 5.71 vs. 1.83 ± 1.10), slower disease progression (14.13 ± 5.54 vs. 3.93 ± 2.12), lower grades of H‐Y(3.13 ± 0.83 vs. 2.20 ± 0.86), and higher frequency of hyperreflexia (75.0% vs. 20.0%) (all *p* < .05) (Table 2). For nonmotor symptoms, the frequency of fatigue was more common in those with *Parkin* gene variants (87.5% vs. 6.7%) with higher olfaction test score (13.71 ± 1.50 vs. 10.33 ± 3.13) (*p* < .01). Data on clinical and demographic characteristics, such as gender (*p* = .193), current age (*p* = .604), UPDRS‐III scores (*p* = .067), dyskinesia (*p* = .221), tremor (*p* = .379), cognition function (*p* = .785), constipation (*p* = 1.000), and levodopa equivalent doses (LEDD) (*p* = .742), were statistically insignificant.

**TABLE 2:** Demographic and clinical data of the enrolled patients with EOPD

Variable	Total subjects (*n* = 23)	With *Parkin* variants(*n* = 8)	Without *Parkin* variants (*n* = 15)	*p*‐Value
Male, *n* (%)	12 (52.2)	6 (75.0)	6 (40.0)	.193
Current age (years)	42.96 ± 8.58	41.25 ± 13.19	43.87 ± 5.13	.604
Age of onset(years)	35.48 ± 8.41	27.13 ± 8.10	39.93 ± 4.15	**.002** *
Delay in diagnosis (years)	4.89 ± 5.43	10.63 ± 5.71	1.83 ± 1.10	**.003** *
Disease duration(years)	7.48 ± 6.10	14.13 ± 5.54	3.93 ± 2.12	**.001** *
H‐Y stage	2.52 ± 0.95	3.13 ± 0.83	2.20 ± 0.86	**.025** *
UPDRS‐III	30.91 ± 16.20	40.14 ± 19.27	26.60 ± 13.10	.067
Dyskinesia, *n* (%)	10 (43.5)	5 (62.5)	5 (33.3)	.221
Tremor, *n* (%)	10 (43.5)	2 (25.0)	8 (53.3)	.379
Hyperreflexia, *n* (%)	9 (39.1)	6 (75.0)	3 (20.0)	**.023** *
Fatigue, *n* (%)	8 (34.8)	7 (87.5)	1 (6.7)	**.000** *
Hyposmia	11.41 ± 3.13	13.71 ± 1.50	10.33 ± 3.13	**.003** *
MMSE	28.68 ± 1.25	28.57 ± 1.62	28.73 ± 1.10	.785
RBD, *n* (%)	2 (8.7)	0 (0)	2 (13.3)	–
Constipation, *n* (%)	11 (47.8)	4 (50.0)	7 (46.7%)	1.000
LEDD	467.61 ± 265.94	439.29 ± 276.55	480.83 ± 269.67	.742
DBS therapy	6/23	2/8	4/15	–

Abbreviations: DBS, deep brain stimulation; EOPD, early‐onset Parkinson's disease; H‐Y, Hoehn and Yahr; LEDD, levodopa equivalent daily dose; MMSE, mini‐mental status examination; RBD, rapid eye movement sleep behavior disorder; UPDRS, unified Parkinson's disease rating scale.

* *p* < .05. Bold indicates the data that had significant difference after pairwise comparison.

## 4 DISCUSSION

Our study identifies the genetic cause for sporadic EOPD (onset age ≤ 45 years) in a Chinese cohort using an integrated genetic approach. Of the 23 patients, 39.1% individuals may be explained by the detected variants. *Parkin* was the most prevalent causative gene in this cohort, since pathogenic/likely pathogenic variants of *Parkin* were identified in 34.8% patients. The detection rate for pathogenic gene variants varies widely among the previous studies, 3.8% in Polish (Koziorowski et al., 2010), 8.6% in UK (Kilarski et al., 2012), 10.8% in Southern Spain (Bandres‐Ciga et al., 2016), 23.1% in India (Pandey et al., 2019), 4.3% in Taiwan (Lin, Chen, et al., 2019), and 7.5% in China (Li et al., 2020). A wide variation was prevalent depending on the studied population, methods used, and the age of the subjects under study. In spite of the relatively small sample size, the efficiency of NGS was used to investigate the frequency of potentially pathogenic known PD genes mutations in EOPD, thus making NGS as an important clinical diagnostic tool in EOPD.

It has been reported that *Parkin* mutations are highly diverse, and they include missense mutations and nonsense mutations, frameshifts, rearrangements with exon deletion or multiplications (Lunati et al., 2018). The systematic MDSGene review showed the following mutation types: the most common structural variants (43.2%) followed by missense mutations in 22.3% and frameshift mutations in 16.5% cases (Kasten et al., 2018). Our data show exon deletions (62.5%) as the most common types of *Parkin* variants, and the locations of these exon rearrangements in the *Parkin* gene were detected in exons 2–9. But, we did not found the deletion of exons 1,10‐12. The results indicate that exon dosage mutations in *Parkin* gene might be the main cause for sporadic EOPD, similar to the reports among other Chinese studies (Guo et al., 2010, 2015; Lin, Zeng, et al., 2019). Tang BS group (Guo et al., 2010) reported that 12.6% of the patients with sporadic EOPD (onset age ≤ 50) carried *Parkin* exon deletions by real‐time quantitative PCR analysis in 2010 and later another study in 2015 revealed 11.4% of EOPD patients (onset age ≤ 45) with *Parkin* exon rearrangements by MLPA assay, deletion being the main mutational cause, especially in exon 2–5 (Guo et al., 2015). A recent study by Lin, Zeng, et al. (2019) also showed exon copy number variations (CNVs) as relatively common in the *Parkin* gene (6.7%). Therefore, exon dosage mutation of *Parkin* is quite common in Chinese EOPD. Traditionally, CNVs were detected by real‐time quantitative PCR or MLPA, and however, recently NGS is useful for CNV detection because coding regions are enriched for causal genetic variation (Kadalayil et al., 2015). In our study, we initially suspected six patients with exon CNVs in the *Parkin* gene, as detected by NGS, which were then further confirmed by MLPA assay. MLPA assay is a practical and effective method for screening large deletions and duplications, particularly in small sample sizes (Wildförster & Dekomien, 2009). Therefore, MLPA is very valuable for establishing exon deletions, especially in patients who are highly suspected to be heterozygous carriers of *Parkin* gene variants or those who are seemingly mutation‐negative after sequencing. Therefore, our results revealed combined applications of the NGS and MLPA as effective methods for EOPD genetic diagnosis.

Clinically, our data showed that compared to EOPD patients without *Parkin* variants, patients with *Parkin* variants had a longer diagnostic delay (10.63 ± 5.71 years). They were often being misdiagnosed as dopa‐responsive dystonia. Unusual presentations, such as lack of tremor, gait disorder, and the involvement of lower limbs, might also be contributing to the diagnostic delay (Ruiz‐Lopez et al., 2019). In addition, hyperreflexia, lack of hyposmia, and RBD might highlight some diagnostic challenges. Even though the cognitive function remains normal, the majority of EOPD patients had long disease duration. Various other phenotypic analyses have drawn similar results (Lohmann et al., 2003; Malek et al., 2016; Sixel‐Doring, Lohmann, Klein, Trenkwalder, & Mollenhauer, 2015; Song et al., 2020), thereby making it difficult for the early diagnosis of *Parkin*‐PD without performing a genetic test. Thus, genetic testing is essential for an accurate diagnosis and distinction between different forms of EOPD.

## 5 CONCLUSION

In conclusion, this study presents a systemic genetic analysis of a Chinese cohort to elucidate the genetic architecture of sporadic EOPD. Our results revealed a higher prevalence of *Parkin* mutations in Chinese patients with sporadic EOPD and emphasized its unique clinical characteristics. Considering the relative small size of the current cohort, studies with larger populations are needed to further elucidate genetic dispositions among Chinese patients.

## CONFLICT OF INTEREST

The authors report no conflicts of interest in this work.

## AUTHORS' CONTRIBUTIONS

Y.J, M.Y, Y.Y, and Z.W are responsible for the conception and design of the study. Y.J, M.Y, J.C, H.Z, W.S, Y.S, F.L, L.W, and Z.W were in charge of data collection and analysis of our study. Y.J drafted the manuscript. Y.Y and Z.W were responsible for the critical revision of the manuscript. E.P and L.Z approved the final version of the manuscript. Y.Y and Z.W should be considered joint senior authors.

## Supporting information

Table S1

Table S2

## Data Availability

The data that support the findings of this study are available on request from the corresponding author.
