# Supplementary material for: Parkin is the most common causative gene in a cohort of mainland Chinese patients with sporadic early‐onset Parkinson's disease
Source: Brain Behav. 2020 Jul 16;10(9):e01765. doi: 10.1002/brb3.1765 (PMC7507393; doi:10.1002/brb3.1765)
Supplement: Supplementary file 1 — Table S1 [file BRB3-10-e01765-s001.docx]

Table S1. One hundred and thirty-six genes included in the extrapyramidal disease panel.

| *MCCC1* | *CRAT* | *REPS1* | *DNAJC12* | *CP* | *MC1R* | *PSEN1* |
| --- | --- | --- | --- | --- | --- | --- |
| *SNCB* | *MAPT* | *TARDBP* | *APTX* | *ATM* | *FA2H* | *UCHL1* |
| *TDP1* | *SLC19A3* | *SPG11* | *VPS13A* | *TIMM8A* | *DCTN1* | *SPR* |
| *ADCY5* | *TOR1A* | *HPCA* | *TUBB4A* | *THAP1* | *SLC2A1* | *SGCE* |
| *PRKRA* | *CACNA1B* | *CIZ1* | *COL6A3* | *GNAL* | *KCTD17* | *ANO3* |
| *KMT2B* | *GCH1* | *DRD5* | *STX1B* | *DRD3* | *SETX* | *FUS* |
| *CHMP2B* | *CACNA1A* | *PRRT2* | *FXN* | *GBA* | *GCDH* | *HMOX1* |
| *HTT* | *SLC30A10* | *SLC39A14* | *SLC18A2* | *SLC6A3* | *TWNK* | *ACTB* |
| *ATP13A2* | *ECM1* | *NDUFS4* | *C19orf12* | *FTL* | *PANK2* | *WDR45* |
| *COASY* | *GRN* | *NPC1* | *SNCA* | *PRKN* | *PINK1* | *PARK7* |
| *LRRK2* | *GIGYF2* | *HTRA2* | *PLA2G6* | *FBXO7* | *VPS35* | *EIF4G1* |
| *DNAJC6* | *SYNJ1* | *DNAJC13* | *CHCHD2* | *VPS13C* | *GLUD2* | *PACRG* |
| *LRP10* | *SNCAIP* | *ADH1C* | *ATP6AP2* | *COQ2* | *POLG* | *ATP1A3* |
| *ATXN1* | *ATXN2* | *ATXN3* | *SPTBN2* | *ZNF592* | *ATXN7* | *ATXN8* |
| *ATXN10* | *TTBK2* | *PPP2R2B* | *KCNC3* | *PRKCG* | *ITPR1* | *TBP* |
| *KCND3* | *PDYN* | *EEF2* | *FGF14* | *AFG3L2* | *TGM6* | *STUB1* |
| *ABCB7* | *ATP2B3* | *TH* | *RAB39B* | *DCAF17* | *ATP7B* | *TAF1* |
| *TENM4* | *DAGLA* | *MYORG* | *NOTCH2NLC* | *DRD2* | *FBXW7* | *GJD2* |
| *HS1BP3* | *MR1* | *NR4A2* | *PLEKHG4* | *RAB29* | *JAM2* | *INPP5F* |
| *PRAF2* | *SIPA1L2* | *TOR1B* |  |  |  |  |
