# Supplementary material for: Parkin is the most common causative gene in a cohort of mainland Chinese patients with sporadic early‐onset Parkinson's disease
Source: Brain Behav. 2020 Jul 16;10(9):e01765. doi: 10.1002/brb3.1765 (PMC7507393; doi:10.1002/brb3.1765)
Supplement: Supplementary file 2 — Table S2 [file BRB3-10-e01765-s002.docx]

Table S2. Demographic and clinical variables of the subjects

| *ID* | Sex | age (years) | AOO  (years) | Disease duration  (years) | Delay in diagnosis (years) | Tremor | Dyskinesia | Hyper  reflexia | H&Y stage | UPDRS-III(score) | Fatigue | Hyposmia  (score) | Constipation | MMSE  (score) | RBD | LEDD  (mg) |
| --- | --- | --- | --- | --- | --- | --- | --- | --- | --- | --- | --- | --- | --- | --- | --- | --- |
| 1 | F | 25 | 19 | 6 | 6 | N | N | - | 2.0 | - | Y | - | - | - | - | - |
| 2 | M | 47 | 30 | 17 | 17 | Y | Y | Y | 4.0 | 49 | Y | 13 | Y | 26 | N | 500 |
| 3 | M | 50 | 34 | 16 | 16 | N | N | Y | 3.0 | 23 | Y | 15 | N | 30 | N | 200 |
| 4 | F | 45 | 37 | 8 | 1 | Y | Y | N | 4.0 | 53 | N | 14 | N | 30 | N | 875 |
| 5 | M | 51 | 33 | 18 | 2 | Y | Y | N | 4.0 | 33 | Y | 15 | Y | 30 | N | 837.5 |
| 6 | M | 44 | 39 | 5 | 1 | Y | Y | N | 2.0 | 44 | N | 12 | N | 30 | Y | 600 |
| 7 | F | 38 | 35 | 3 | 3 | N | N | N | 1.0 | 8 | N | 12 | Y | 28 | N | 200 |
| 8 | M | 40 | 39 | 1 | 0.5 | N | N | N | 1.0 | 25 | N | 11 | N | 29 | N | 300 |
| 9 | M | 18 | 13 | 5 | 4 | N | N | Y | 2.0 | 32 | Y | 15 | Y | 30 | N | 200 |
| 10 | F | 50 | 45 | 5 | 1 | Y | N | Y | 3.0 | 27 | N | 8 | Y | 29 | N | 312.5 |
| 11 | M | 46 | 43 | 3 | 2 | N | N | N | 2.0 | 24 | N | 5 | N | 27 | N | 600 |
| 12 | F | 34 | 31 | 3 | 1 | N | Y | Y | 3.0 | 27 | N | 13 | Y | 30 | Y | 550 |
| 13 | M | 46 | 43 | 3 | 3 | Y | N | N | 2.0 | 35 | N | 12 | Y | 29 | N | 287.5 |
| 14 | M | 46 | 44 | 2 | 2 | Y | N | N | 3.0 | 22 | N | 6 | N | 30 | N | 350 |
| 15 | F | 46 | 42 | 4 | 1 | Y | N | N | 2.0 | 29 | N | 9 | Y | 28 | N | 675 |
| 16 | F | 39 | 36 | 3 | 3 | Y | N | N | 2.0 | 7 | N | 15 | N | 29 | N | 175 |
| Table S2. Demographic and clinical variables of the subjects | | | | | | | | | | | | | | | | |
| ID | Sex | age (years) | AOO  (years) | Disease duration  (years) | Delay in diagnosis (years) | Tremor | Dyskinesia | Hyper  reflexia | H&Y stage | UPDRS-III(score) | Fatigue | Hyposmia  (score) | Constipation | MMSE  (score) | RBD | LEDD  (mg) |
| 17 | M | 37 | 36 | 1 | 1 | N | N | N | 1.0 | 17 | N | 8 | N | 29 | N | 0 |
| 18 | F | 52 | 44 | 8 | 1 | N | Y | Y | 3.0 | 40 | N | 6 | Y | 27 | N | 737.5 |
| 19 | M | 51 | 31 | 20 | 12 | N | Y | N | 4.0 | 71 | Y | 14 | N | 29 | N | 700 |
| 20 | F | 37 | 22 | 15 | 11 | Y | Y | Y | 3.0 | 56 | N | 13 | N | 27 | N | 112.5 |
| 21 | F | 48 | 43 | 5 | 3 | N | Y | N | 2.0 | 31 | N | 11 | N | 29 | Y | 750 |
| 22 | M | 51 | 35 | 16 | 16 | N | Y | Y | 3.0 | 17 | Y | 11 | N | 28 | N | 525 |
| 23 | M | 47 | 42 | 5 | 4 | Y | N | N | 2.0 | 10 | N | 13 | Y | 27 | N | 900 |

(Abbreviations: M: male; F: female; H-Y: Hoehn and Yahr; UPDRS, unified Parkinson’s disease rating scale; MMSE, mini-mental scale evaluation; RBD, rapid eye movement behavior disorder; LEDD, levodopa equivalent daily dose ;DBS, deep brain stimulation.)
